# Primary Care Behavioral Health in Sweden – a protocol of a cluster randomized trial evaluating outcomes related to implementation, organization, and patients (KAIROS)

**DOI:** 10.1186/s12913-023-10180-9

**Published:** 2023-10-31

**Authors:** Anneli Farnsworth von Cederwald, Josefine L. Lilja, Nils Hentati Isacsson, Viktor Kaldo

**Affiliations:** 1 Department of Psychology, Faculty of Health and Life Sciences, Linnaeus University, Växjö, Sweden; 2 School of Public Health and Community Medicine, Institute of Medicine, Sahlgrenska Academy, University of Gothenburg, Gothenburg, Sweden; 3 Närhälsan Research and Development Primary Health Care, Region Västra Götaland, Gothenburg, Sweden; 4 Centre for Psychiatry Research, Department of Clinical Neuroscience, Karolinska Institutet, & Stockholm Health Care Services, Region Stockholm, Stockholm, Sweden

**Keywords:** PCBH, Primary care behavioral health, Primary care, Integrated care, Behavioral health, Mental health, Implementation, Cluster randomization

## Abstract

**Background:**

Providing comprehensive and continuous care for patients whose conditions have mental or behavioral components is a central challenge in primary care and an important part of improving universal health coverage. There is a great need for high and routine availability of psychological interventions, but traditional methods for delivering psychotherapy often result in low reach and long wait times. Primary Care Behavioral Health (PCBH) is a method for organizing primary care in which behavioral health staff provide brief, flexible interventions to a large part of the population in active collaboration with other providers. While PCBH holds promise in addressing important challenges, it has not yet been thoroughly evaluated.

**Methods:**

This cluster randomized trial will assess 17 primary care centers (PCCs) that are starting a PCBH implementation process. The PCCs will be divided into two groups, with one starting immediate implementation and the other acting as a control, implementing six months later. The purpose of the study is to strengthen the evidence base for PCBH regarding implementation-, organization-, and patient-level outcomes, taking into consideration that there is a partially dependent relationship between the three levels. Patient outcomes (such as increased daily functioning and reduction of symptoms) may be dependent on organizational changes (such as availability of treatment, waiting times and interprofessional teamwork), which in turn requires change in implementation outcomes (most notably, model fidelity). In addition to the main analysis, five secondary analyses will compare groups based on different combinations of randomization and time periods, specifically before and after each center achieves sufficient PCBH fidelity.

**Discussion:**

A randomized comparison of PCBH and traditional primary care has, to our knowledge, not been made before. While the naturalistic setting and the intricacies of implementation pose certain challenges, we have designed this study in an effort to evaluate the causal effects of PCBH despite these complex aspects. The results of this project will be helpful in guiding decisions on how to organize the delivery of behavioral interventions and psychological treatment within the context of primary care in Sweden and elsewhere.

**Trial registration:**

ClinicalTrials.gov: NCT05335382. Retrospectively registered on March 13th, 2022.

**Supplementary Information:**

The online version contains supplementary material available at 10.1186/s12913-023-10180-9.

## Background

Primary care constitutes a foundation for the healthcare system by providing a first line of somatic and mental care, as well as continuous, long-term management of chronic and complex health problems [1]. Almost half (46%) of all adults experience mental illness over the course of their lifetime, and the majority of these patients are treated in primary care, where up to a third of all visits are related to mental health [2–4]. Mental health conditions substantially reduce functioning [5] and quality of life [6], as well as contributing significantly to sickness absence, productivity loss and unemployment [7, 8]. In addition, there is a strong relationship between mental health and chronic somatic diseases, such as diabetes, obesity, and persistent pain, and many patients have complex health problems with multiple somatic and mental comorbidities as well as social difficulties [9–11]. In addition to the suffering experienced by the individual, these conditions pose an enormous economic burden [12]. It therefore follows that the quality of primary care has direct implications for many patients’ health and daily lives, as well as society at large. As such, the World Health Organization (WHO) has designated the integration of mental health services into primary care a key objective, highlighting its role in achieving universal health coverage [13, 14]. However, the volume and complexity of mental health concerns presenting in primary care currently exceeds its capacity and competence [13, 15, 16]. In Sweden, most primary care centers (PCCs) have introduced on-site clinical psychologists or social workers, and national policies state that early access to psychological treatments is to be prioritized over pharmacological interventions in most cases of mild to moderate mental health problems [17, 18]. However, in many PCCs psychological care is still hard to access, likely because the traditional model for delivering psychotherapy and psychosocial interventions is impossible to scale up enough to meet the need for such treatment [19]. Consequently, Swedish primary care patients rarely receive nonpharmacological interventions for their mental and behavioral health, with only 3% of patients meeting with a psychologist or social worker [20]. On a global scale, the service gap is even more pronounced, with a large proportion of people with mental health conditions receiving no formal care at all [21].

Primary Care Behavioral Health (PCBH) is a team-based model aiming to enhance primary care by integrating behavioral health expertise, with the goal of improving care for the entire clinic population [22]. Interventions within the model focus on behaviors and skills relevant for preventing deleterious somatic and psychological health outcomes and can take many forms, for example as selected interventions from evidence-based cognitive behavioral therapy (CBT) manuals or as interventions specifically designed to be brief, such as Focused Acceptance and Commitment Therapy (FACT) [16, 23–26]. PCBH is characterized by routine delivery of such interventions to a large proportion of patients, with high availability and productivity. It utilizes teamwork and task-sharing between and across professional disciplines to increase flexibility, efficiency, and treatment adherence as well as share knowledge and build consensus among staff. A central part of the model is freeing up resources by providing brief visits, usually no more than 30 min in length, one visit at a time rather than in a planned series of visits. Follow-up is based on a ‘consultant’ rather than a ‘therapist’ approach, where the patient is not followed until the point of remission but until there is some improvement in symptoms or functioning. Treatments are open-ended, meaning that patients can easily book another visit if they need to. For large or resource-intensive patient categories, clinical pathways incorporating group sessions or online interventions are frequently employed. A pragmatic and population-based mindset, where the available resources are used to reach as many patients as possible, permeates the model.

PCBH has gained popularity, likely due to its promise of a much-needed increase in the availability of psychological interventions. Previous research indicates that brief psychotherapeutic interventions within integrated care lead to broad improvements in symptom reduction, functioning and well-being across a multitude of problem areas [23, 25, 27–41]. Studies have also indicated that PCBH is cost-effective [41–47], appreciated by care providers [45, 48–51] as well as patients [48, 50, 52], and that the model can improve access to and reduce wait time for mental health services [53, 54]. However, as of yet, there are no large-scale comparative studies of high quality [55, 56]. Achieving and studying change in primary care can be difficult, especially when implementing complex interventions that require change across multiple levels [57]. This is especially true for PCBH, as most mental health staff lack training in how to effectively adapt to the primary care setting, which complicates implementation and highlights the role of model fidelity in the evaluation of the model [58–60].

## Methods/design

**Fig. 1:**
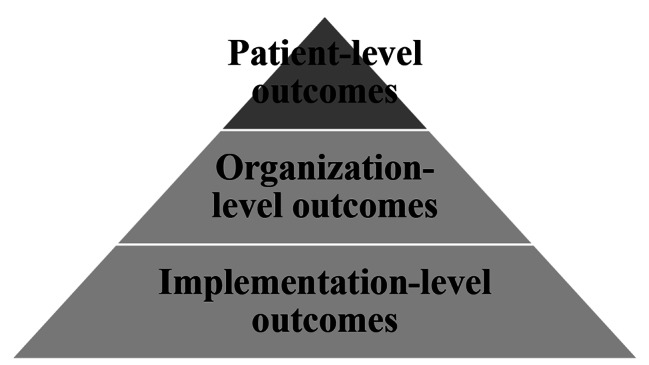
Dependent relationships between levels of outcomes

The main purpose of the current project is to strengthen the evidence base for PCBH regarding implementation-, organization-, and patient-level outcomes by conducting the first large-scale randomized study on the model, taking a step toward answering whether the effects of the model are large enough to merit scaling up implementation. The research questions of primary interest are divided into three levels, where outcomes of higher levels are more or less dependent on the lower levels (i.e., patient outcomes may be dependent on organizational outcomes and both are clearly dependent on implementation outcomes; see Fig. 1):

- Implementation level:
  - What is the timeline for partial implementation (FID2) as well as achieving adequate model fidelity (FID3, primary implementation level outcome) in PCCs adopting the PCBH model?
  - How do healthcare staff perceive the acceptability, feasibility, and appropriateness of PCBH, and to what extent do they adhere to the model?
  - What are the observed obstacles and facilitators during implementation?
- Organizational level:
  - Is PCBH superior to traditional Swedish primary care in reducing waiting times (primary organization level outcome) and increasing patient reach?
  - Does PCBH result in shorter treatment lengths and lower costs for staff and resources compared to traditional primary care?
  - Does PCBH improve interprofessional collaboration and staff work environment compared to traditional care?
  - Does PCBH impact medication prescription patterns, sick leave utilization, and patients’ care consumption, thereby influencing related costs?
- Patient level:
  - Is PCBH superior to traditional Swedish primary care in terms of patient outcomes, including everyday functioning (primary patient level outcome), symptoms, quality of life, satisfaction with care, subjective change, and adverse events?
  - If not superior, is PCBH non-inferior to traditional primary care?
  - What methods are used within brief interventions and what strategies do patients adopt after treatment?

The primary comparisons between PCBH and traditional primary care will consider model fidelity at both the organizational and patient levels. The main analysis will compare PCCs randomized to the early implementation arm once they reach satisfactory fidelity with PCCs in the control group.

### Design and overall procedure

The project is a cluster-randomized clinical trial following Good Clinical Practice (GCP) principles. Standard Operating Procedures will be developed for key routines, and digital Client Report Files will be stored in the study technical platform provided by Karolinska Core Facilities. Study coordinators will primarily monitor data quality and routines internally. This protocol was devised according to the SPIRIT reporting guidelines [61]. Any substantial amendments to this protocol will be tracked, dated and described in trial reports.

Data will be collected at Närhälsan, the provider of public primary care in Västra Götaland, Sweden, consisting of 104 PCCs in both urban and rural areas. This project studies a naturalistic implementation effort initiated by Närhälsan, with external trainers and supervisors procured by the organization. The study will utilize a cluster-randomized design, randomly assigning PCCs to either implement PCBH immediately (EARLY) during the first 5–9-month period or act as control centers and implement PCBH during the second period (DELAYED). Period 1 starts when all PCCs in the EARLY group have scheduled starting dates during on-site consultations. Period 2 begins when all PCCs in the DELAYED group have done the same, within 5 to 9 months after the start of Period 1. Provided sufficient project resources, period 2 can be lengthened to allow for continued data collection. Patient measures will be collected up to two months before Period 1, and some organizational measures up to one year in advance, establishing a baseline for each PCC. As the project, due to the naturalistic approach, has little direct control over how well a PCC implements PCBH, the analysis plan covers several scenarios where varying degrees of model fidelity are considered (see Data analysis plan).

### Participating primary care centers and randomization

The project includes 8 subregions within Närhälsan, each providing 1–4 PCCs that do not currently adhere to PCBH principles but are willing to implement the model and be part of the randomization process. No other inclusion or exclusion criteria were applied. Basic characteristics of PCCs will be collected through semi-structured interviews at the beginning of the study. This includes information such as the number of listed patients, sociodemographic indexes, number of mental health staff and their training, whether there is and the size of any queues for mental health treatment, as well as details about triage, referrals, and teamwork routines.

In total, 17 PCCs serving a total of 187 000 patients have chosen to participate. For a list of sites, see ClinicalTrials.gov registration. Cluster randomization between the EARLY and DELAYED arms will be conducted using the online tool randomization.com in a 1:1 ratio. The Karolinska Trial Alliance (KTA), an independent unit, performs the randomization separately for each subregion without block division to ensure at least one PCC in the EARLY arm per subregion. If the distribution of PCCs between study arms deviates by more than one, KTA will repeat the randomization until the distribution is balanced or deviates by one. Only the results will be shared with the researchers. Any additional PCCs joining the project after subregion randomization but before the start of implementation period 1 will be randomized by KTA using list randomization with even blocks of random sizes determined by KTA and not shared with researchers.

### Participant recruitment and power analysis

All patients aged 18 and above who are suitable for behavioral or mental health interventions, regardless of diagnoses and concomitant care, will be invited to participate in the study. This inclusive approach aligns with the naturalistic setting and the broad scope of the PCBH model. Trained research assistants will identify and contact potential participants, providing digital access to written information and a consent form. Participants will be kept blind to the implementation status of PCBH in their respective PCCs, as all are only informed that the study aims to evaluate and improve mental health treatment in primary care.

We estimate that 70% of patients identified and informed about the study will consent to participate. Power calculations aiming for a power of 90% in detecting a difference of *d* = 0.4 on a superiority test on the primary patient outcome, adjusted for the lower power in a cluster randomized design with 8 PCCs in one group and 9 in the other, estimating the Intracluster Correlation Coefficient (ICC) to 0.01 [62] and a follow-up response rate of 80%, indicate that a total of 476 patients need to be analyzed, 595 patients recruited, and 850 identified and informed.

If PCBH’s superiority over traditional care is not established, a secondary aim is to determine if PCBH is non-inferior. Since there is currently no empirically based definition of the minimal clinically important difference for WHODAS-12 [63] and even less so for the 8-item version we use (see below), we will adopt a conservative non-inferiority margin based on recommendations for a minimally relevant clinical effect for depression, with a Cohen’s *d* of 0.24 [64]. To achieve 80% power with the same ICC as mentioned earlier, this analysis would require the inclusion of 815 patients, with a total of 1455 patients identified and informed. However, this is a secondary objective dependent on resource availability. In case the expected rates of inclusion or follow-up responses are lower than anticipated, we will consider extending the implementation periods to increase participant enrollment.

### Participating primary care staff

Primary care staff members including physicians, nurses, mental health staff, and management at each PCC will be provided with written information about the study. Those who agree to participate will complete questionnaires assessing work environment and PCBH implementation aspects and may be invited to participate in focus groups.

### Measures and assessments

Measures and assessments are categorized into implementation, organization, and patient levels. These will be collected through administrative systems, medical records, national registers, staff and patient self-report forms (primarily via a digital platform, via phone when needed) as well as semi-structured interviews conducted by independent research personnel. Table 1 provides an overview of all patient-level self-report questionnaires and timepoints. To enhance participant retention, automated text message reminders are sent for each assessment. Trained research assistants will also proactively contact patients who have not completed their assessments via phone. For participants who wish to discontinue due to time constraints, the option of completing a shorter assessment that includes the most important questionnaires will be provided. As a final option, they will be given the opportunity to complete only the primary patient outcome assessment over the phone.

### Implementation outcomes

#### Overall categorical PCBH model fidelity

To assess fidelity, implementation measures (see Model adherence, obstacles, and facilitators) and semi-structured interviews with PCC managers will be used. Three fidelity levels are established: FID1 represents no PCBH implementation, FID2 represents partial PCBH implementation, and FID3 represents adequate PCBH implementation. Two of the authors (AFvC, JLL), along with independent PCBH experts and supervisors, will determine the timepoints when each PCC transitions between fidelity levels. The primary outcome is the time taken for a PCC to reach FID3 level. Secondary outcomes include whether a PCC reaches FID3 within the preset implementation period and the time taken to reach FID2 level. These categories will be used as a base for some analyses, including the primary analysis for organizational and patient level data (see Data Analysis Plan).

#### Implementation success

The s-NoMAD questionnaire, which evaluates the integration of complex interventions into daily practice [65], will be administered to nurses, physicians, mental health workers, and leaders to assess implementation success.

#### Acceptability, appropriateness and feasibility

The Acceptability of Intervention Measure (AIM), Intervention Appropriateness Measure (IAM), and Feasibility of Intervention Measure (FIM) [66] are four-item measures of implementation outcomes that are considered particularly relevant to implementation success [67]. Profession-specific versions of these will be administered to nurses, physicians, mental health workers and leaders, focusing on key elements of the PCBH model that are particularly relevant to their daily work.

#### Model adherence, obstacles, and facilitators

- Average Appointment Length (AAL) assesses the average duration of bookable appointments in mental health workers’ schedules, measured in minutes.
- Supply of Appointments (SA) measures the number of bookable appointments available per full-time mental health worker on a weekly basis and reflects the capacity and availability of PCBH services.
- Future Capacity (FC) indicates the number of available timeslots for appointments in the coming 4 weeks divided by the total number of timeslots in mental health staff schedules. It provides insights into the PCC’s ability to accommodate new patients.
- Percentage Longer Treatments (PLT) indicates the proportion of patients who have exceeded the standard number of visits (more than 4), signaling a departure from usual PCBH routine.
- The Primary Care Behavioral Health Provider Adherence Questionnaire (PPAQ) [68] assesses the adherence of behavioral health providers to the PCBH model. For this study, it was adapted for the Swedish setting, incorporating forward and backward translation, the addition of new items, and the removal of irrelevant items. To address the teamwork aspect of PCBH, similar questionnaires were developed for physicians and nurses in collaboration with Swedish PCBH experts, utilizing item-level Content Validity Index ratings [69]. The resulting scales are named Integrated Behaviors in Primary Care for General Practitioners / Mental Health / Registered Nurses (IBPC-GP, -MH, -RN).
- Levels of Integration Measure (LIM) [70] evaluates healthcare leaders’ perception of the PCC’s integration level regarding behavioral health staff. Similar to the PPAQ, adaptations have been made to reflect its use in the Swedish healthcare system.

In addition to these measures, PCBH supervisors will provide ratings and structured qualitative descriptions of each PCC’s progress in the implementation process after every supervision meeting. This feedback will capture the supervisors’ perspectives on obstacles and facilitators encountered during the local implementation processes.

.

### Organizational outcomes

#### Measures relevant to patient reach

The penetration rate indicates the number of unique patients seen per full-time equivalent mental health professional and will be measured in relation to all listed patients (PR1) as well as patients with a mental health diagnosis (PR2).

#### Work environment and teamwork among staff

To evaluate any changes in staff work environment, 35 items from the Copenhagen Psychosocial Questionnaire (COPSOQ-III) [71], a measure of psychosocial work environment, will be administered to participating staff. The subscales of Consensus and Collaboration of the IBPC-GP, -RN and -MH scales will be used to measure teamwork at the PCC.

#### Measures relevant of access of care and wait times

- Average waiting time (AWT, primary organizational outcome) captures the waiting time from the identification of a behavioral health concern to being seen by any clinician. It includes the time between the patient’s initial contact (phone call or message) and the visit with any clinician. If the concern is identified during another visit, the wait time is calculated from the visit where the need was identified to the visit with the referred-to profession.
- Average waiting time for a psychosocial intervention (AWT-P) focuses specifically on the waiting time from the identification of a behavioral health concern to receiving any mental health intervention.
- Number of individuals on waiting lists to receive psychosocial interventions (WL).
- Third Next Available Appointment (TNAA) assesses the waiting time until the third next available appointment for a hypothetical patient who contacts the PCC on a given day. It is measured consistently at the same time every week (e.g., Mondays at 9 AM). TNAA provides insights into the access and availability of care, independent of patients’ own ability to show up at a given time.
- Average number of visits and phone/video contacts during patients’ period of care.

#### Cost, health economics and utilization of medication and sick leave

Utilization of medical care and productivity losses will be measured using the Trimbos and iMTA questionnaire on Costs associated with Psychiatric Illness (TiC-P). This includes assessing the number of days on sick leave, productivity loss while at work, productivity loss at home, and medications prescribed and taken. The 5-item Eq. 5D will be administered for health economic evaluations. Cancelled Visits and No-Shows (CVNS) will be tracked, capturing the number of visits where patients either cancelled late or did not show up. Specialist Referrals (SR) will be recorded as the percentage of patients referred to specialized care and Denied Referrals (DR) will indicate how many of these referrals were not accepted. Patient self-reports will be complemented and combined with national register-level data on sick leave, medications, and health care utilization.

### Patient outcomes

The main time-points for all non-register patient measurements are as follows:

- Before or shortly after first contact with the PCC. This is considered the pre-measure timepoint.
- 4-week follow-up (FU4).
- 8-week follow-up (FU8).
- 12-week follow-up (FU12). This timepoint is considered post-measure when applicable.
- One year follow-up (FU52).

#### Sociodemographic and clinical data

Social and economic status, education, profession, and employment/activity status will be collected through self-report. The main reason for the visit, the duration of the problem, other health concerns, concomitant care, care consumption, ICD-10 diagnoses and medications will be captured through self-reports and medical records.

#### Everyday functioning, symptoms, and quality of life

The primary patient outcome is everyday functioning, assessed through four domains (8 out of 12 items) of the WHO Disability Assessment Schedule 2.0 (WHODAS-12) [72]: Life activities, Cognition, Getting along, and Participation. The domains of Mobility and Self-care, which form independent factors and are not expected to change significantly, will be excluded from primary analyses but considered in secondary analyses. A range of very short versions of well-established patient-rated scales for common mental health conditions will be used to measure symptoms. For an overview, see Table 1. Each scale will be used to evaluate changes to the specific symptoms measured, as well as for dividing patients into different analysis groups based on problem areas. Additionally, a total index for symptom load will be calculated from all 21 items, with appropriate weighting to account for the varying number of questions across domains. Quality of Life will be measured by the 12-item Brunnsviken Brief Quality of Life (BBQ) [73], which examines the importance and fulfilment of six areas (e.g. spare time quality, creative work, and friendship). Additionally, the 4-item Outcome Rating Scale (ORS) [74] will be used. Complementing these measures is the DAily Routines for Well-being INventory (DARWIN), developed by the research group, which evaluates the frequency of 11 behaviors relevant to mental health, including exercise, regular eating, socializing, and sleep hygiene.

**Table 1:** Overview of patient self-report questionnaires measuring symptoms, including timepoints

Questionnaires measuring symptoms	Initial visit	FU4	FU8	FU12	FU52
Patient Health Questionnaire 9-item (PHQ-9)^1^	X			X	X
Generalized Anxiety Disorder 7-item (GAD-7)^1^	X			X	X
Patient Health Questionnaire 4-item (PHQ-4)^1^		X	X		
Panic Disorder Severity Scale 2-item (PDSS-SR-2)^2^	X	X	X	X	X
Social Phobia Inventory – Abbreviated Version (Mini-SPIN-3)^1^	X	X	X	X	X
Obsessive Compulsive Disorder 3-item (OCD-3)^3^	X	X	X	X	X
Perceived Stress Scale 2-item (PSS-2)^1^	X	X	X	X	X
One-item question on perceived stress^3^	X	X	X	X	X
Karolinska Exhaustion Disorder Scale 3-item (KEDS-3)^3^	X	X	X	X	X
Insomnia Severity Index 2-item (ISI-2)^1^	X	X	X	X	X
Short Health Anxiety Inventory 3-item (SHAI-3)^2^	X	X	X	X	X
The Alcohol Use Disorders Identification Test-Concise (AUDIT-C-3)^1^	X	X	X	X	X
Pain (One-item rating from 0 to 10)^3^	X	X	X	X	X

^1^ Published and empirically tested.

^2^ Created by factor analytic item-reduction and sensitivity to change analyses on large datasets from previous trials conducted by the research group, not yet published.

^3^ Created by an expert group, not yet published or empirically tested.

#### Change, adverse events and satisfaction with care

Patient experiences and perceptions will be assessed using multiple measures. Satisfaction will be evaluated using 4 items from the Client Satisfaction Questionnaire (CSQ) [75], the Session Rating Scale (SRS) [76], and 9 additional items specifically designed for PCBH settings to gauge patients’ perceptions of and attitudes toward their care providers. To measure subjective change, the one-item Patient Global Impression - Improvement (PGI-I) [77] scale will be employed. To assess treatment side effects, an Adverse Events questionnaire will be employed, offering both a concise (3 items) and a more extensive version (9 items) at different timepoints, in which patients will have the opportunity to report and describe any unwanted treatment effects or events they may have experienced. Additionally, at the FU12 timepoint, patients will be offered the opportunity to participate in semi-structured interviews to provide detailed insights on their satisfaction, adverse events, treatment content, goals, and perceived changes in symptoms and quality of life. Any adverse events that require immediate action will be reported to the responsible PCC.

#### Treatment content

Information on which interventions are used within each primary care model will be obtained through medical records. In addition, patients will be asked in interview form what interventions they remember and which strategies they use after treatment.

### Primary care models

#### Care as usual (CAU)

CAU refers to the currently most common way of organizing behavioral and psychological care in Sweden. In accordance with this, all CAU centers, in this study the DELAYED arm, will have on-site clinical psychologists or social workers delivering a variety of talking treatments. Crucially, these functions will not be integrated into the PCC’s workflow but will function as co-located mental health services, in line with the traditional model for delivering psychosocial interventions [19]. Typical routines at CAU centers will involve written referrals from physicians, no direct triage to mental health services and caregivers managing their own schedules and bookings. Interdisciplinary collaboration, if present, is typically done only through scheduled team conferences. Productivity as well as the reach of interventions in relation to mental health is ordinarily low, resulting in extensive wait times and queued referrals. The content of psychological treatment within the CAU model will vary depending on the staff present at each PCC, from supportive counseling to manualized CBT and long-term psychodynamic psychotherapies. The actual content of an individual patient’s care will be assessed retroactively through medical records. Semi-structured interviews with PCC managers will be used to assess that no PCCs in the CAU condition have workflows that are reminiscent of PCBH.

#### Primary Care Behavioral Health (PCBH)

An obstacle to high quality PCBH research is that there is a variation in the understanding of the aim and key strategies of the model [59]. In this project, a version of the PCBH model adjusted for the Swedish health care system [15] will be used. The focus of the implementation effort is to increase access and decrease wait times for mental health interventions as well as increase collaboration between psychologists/social workers and medical staff. The planned changes include introducing direct triage, same-day appointments, clinical pathways, and a stepped care model for interventions as well as shortening appointment lengths and increasing productivity. Importantly, the typical PCBH routine of ‘warm hand-offs’ (patients being offered a visit with a mental health professional directly after a visit with a primary care physician) [22] will not be emphasized, as Swedish health care increasingly leans on direct triage, where the physician visit is postponed or replaced entirely [78]. Another aim is to increase the efficiency of interprofessional collaboration by defining and clarifying the role of the mental health staff, educating other professions in when and how to utilize their services as well as introducing a common language and mindset for discussing mental health issues with both patients and colleagues. Routine coordination, efforts to reach a shared understanding among staff and quick ‘curbside consultations’ will be emphasized over scheduled team conferences. Mental health staff will be taught to assess patients’ problems through a contextual rather than a medical model of mental health and administer brief interventions adapted to the patients’ problem and life situation, as described in the introduction. All mental health staff will receive three days of training followed by six 4-hour supervision sessions over the course of six months. Training and supervision will focus on both organizational and clinical aspects of the model. Additional support for individual PCCs will be considered in the case of low model fidelity. A local implementation group will be established at each PCC, consisting of all mental health staff, managers as well as representatives for physicians, nurses, and other relevant occupations. All other staff will receive 2 hours of training. All training and supervision will be conducted by an external part. One of the authors (JLL) will together with research assistants oversee adherence by monitoring implementation outcomes (see Model adherence, obstacles, and facilitators) and giving feedback and recommendations to PCC managers and PCBH supervisors. No additional auditing is planned.

### Data management, monitoring and confidentiality

Data will primarily be collected through and stored in encrypted form in the study technical platform and database (BASS4) provided by Karolinska Core Facilities. Patients and staff fill in questionnaires online. Data are then stored behind two-factor verification and accessible only to selected research personnel. Data collected through means other than self-report forms will be manually entered into BASS4 by research assistants. All data entry is done in premade templates, ensuring that data are in a proper format and within an expected range of values. Any data coding is done according to standardized coding practices. When data collection is finished, all data will be pseudonymized and key coded. Only the principal investigator and university archivists will be able to access the key. Other project team members will only be able to access pseudonymized data. Considering the nature of the trial, which primarily involves data collection from routine clinical practice and presents a low anticipated risk to participants and given that data monitoring for psychological intervention trials is not mandatory in Sweden, we decided to forgo a data monitoring committee due to costs.

### Data analysis plan

We are planning to conduct one main analysis for each of the primary outcomes at each level, a range of explorative and longitudinal analyses for the implementation-level outcomes, and several secondary analyses for the organizational- and patient-level outcomes. Primarily, we aim to confirm that the EARLY arm reaches adequate fidelity level faster than the DELAYED arm and to capture the randomized controlled effects of PCBH on organization- and patient-level outcomes when it has reached adequate model fidelity during the first implementation period (corresponding to an implemented-per-protocol perspective). We also want to evaluate the controlled effects of intending to implement PCBH regardless of what level of fidelity each PCC reaches (corresponding to an intent-to-implement perspective) and the non-controlled (i.e., not comparing the randomized groups) relation between reaching adequate model fidelity and outcomes (corresponding to an adherence-effect or dose-response perspective). No interim analyses are planned.

Implemented-per-protocol analyses:

1. PCCs in EARLY, from when they have reached FID3, compared to all PCCs in DELAYED, during period 1 (main analysis).
2. As (1), but also including data from period 2 in EARLY (but not in DELAYED), to control for the event of PCCs needing extended time to reach FID3, thus lowering the power of the main analysis.

Intent-to-implement analyses:

3. All PCCs regardless of model fidelity in EARLY compared to all PCCs in DELAYED during period 1.
4. As (3), but for periods 1 and 2.

Adherence-response analyses (not controlled by randomization):

5. All PCCs, regardless of arm, when in FID3 during period 1 or 2, compared to PCCs in DELAYED during period 1.
6. As (5), but compared to all PCCs before they reach FID3.

#### Sensitivity analyses

To control for the lack of an objective cutoff for reaching adequate PCBH, sensitivity analyses of the above tests will be conducted where both FID2 and FID3 are used to define adequate PCBH. In addition, sensitivity analyses will also be performed to manage possible inherent differences in the timing of patient pre-measures. Due to the sometimes fast-paced nature of primary care and the naturalistic study design, patients will at times fill in pre-treatment self-report forms after a first visit rather than before. As such, sensitivity analyses will be conducted to see if the temporal relation between pre-treatment measures and initial visit influences the result.

#### Statistical tests

Hierarchical Linear Models and Generalized Estimating Equations will be the primary statistical methods used. Missing data will be addressed through appropriate imputation methods that align with the missing data mechanism and account for the multilevel structure of the data. Alternatively, statistical techniques capable of handling missing data, such as multilevel models, will be employed for analysis. The analyses will take into account the multilevel structure of the data, e.g. nesting patients under PCC for patient outcomes. For Health Economic Analyses, effectiveness will be estimated by the construction quality-adjusted life-years (QALYs) based on EQ-5D. Incremental cost-effectiveness ratios (ICERs) will be computed and Monte Carlo simulation with non-parametric bootstrapping will be performed to account for the uncertainty of the ICER point estimates. Cost-effectiveness acceptability curves (CEACs) will be used to explore cost-effectiveness under varying willingness to pay-circumstances.

## Discussion

The main purpose of the current project is to strengthen the evidence base for the effects that PCBH has on implementation-, organization-, and patient-level outcomes. Implementation of a primary care model such as PCBH needs to be studied in a naturalistic setting, which reduces the possibility for rigorous research procedures and high levels of control. In addition, implementing PCBH is a large undertaking that requires systematic changes on both an organizational and an individual level – processes that are highly dependent on the local context and difficult to control. The implementation in this study is initiated by an external stakeholder, Närhälsan, and performed by a publicly procured company. As such, we as researchers have little control over the specifics of training and supervision, as well as over which PCCs participate in the project, how these are chosen, and their internal contexts and readiness for change. This means that the model fidelity categorization is an essential aspect – especially as many measurements and analyses are hierarchically dependent on each other. Patient outcomes (such as everyday functioning and satisfaction with care) may be partly dependent on organizational outcomes (such as waiting time until first visit) and they are both dependent on implementation outcomes (such as acceptance of and adherence to PCBH routines).

We have designed this study in an effort to evaluate the causal effects of PCBH despite these complex aspects. We have identified relevant measures and divided them into the three hierarchical levels described above. The data analysis plan has been designed to primarily utilize the randomized controlled design from both a per-protocol perspective (focusing on PCCs with successful implementation) and an intent-to-implement perspective (focusing on all PCCs in the EARLY implementation group regardless of which model fidelity category they reach). These analyses are complemented by secondary analyses based only on model fidelity and its association with outcomes, ignoring the randomized design but providing an opportunity of an equivalent of a dose-response analysis. Finally, in case we do not establish superiority for PCBH on the patient-level primary outcome, we will also execute non-inferiority analyses. In total, this results in a large number of planned analyses, but we deem this to be necessary to manage the complexities described above.

The results of this project will be helpful in guiding decisions on how to organize the delivery of behavioral interventions and psychological treatment within the context of primary care in Sweden and elsewhere. As the project is carried out within regular healthcare the external validity is very high, with only a minor gap between research and practice.

### Current trial status

Implementation period 1 started in January 2022. Patients will be included until December 31st 2023, and the last participant is expected to reach the final endpoint (one-year follow-up) in December 2024. Data analysis will begin in May 2024.

### Electronic supplementary material

Below is the link to the electronic supplementary material.

Supplementary Material 1

Supplementary Material 2

Supplementary Material 3

## Data Availability

Data are available upon reasonable request, subject to the requirement that approval from the involved health care services may be necessary for each separate request. Due to the sensitive nature of the data, only fully anonymized data can be made available. Requests can be made to ORCID iD 0000-0002-6443-5279. The approved ethics application is available in Swedish upon request without any conditions.

## Abbreviations

CAU: Care As Usual
FID1, -2, -3: Model Fidelity Category 1, 2, 3
KTA: Karolinska Trial Alliance
PCBH: Primary Care Behavioral Health
PCC: Primary Care Center
