# Supplementary material for: Primary Care Behavioral Health in Sweden – a protocol of a cluster randomized trial evaluating outcomes related to implementation, organization, and patients (KAIROS)
Source: BMC Health Serv Res. 2023 Oct 31;23:1188. doi: 10.1186/s12913-023-10180-9 (PMC10619326; doi:10.1186/s12913-023-10180-9)
Supplement: Supplementary file 1 — Supplementary Material 1 [file 12913_2023_10180_MOESM1_ESM.docx]

**APPENDIX 1**

All items from the World Health Organization Trial Registration Data Set

| **Data category** | **Information** |
| --- | --- |
| Primary registry and trial identifying number | ClinicalTrials.gov: [NCT05335382](https://clinicaltrials.gov/show/NCT05335382) |
| Date of registration in primary registry | 19 April, 2022 |
| Secondary identifying numbers | 2020-04198-A1 |
| Source(s) of monetary or material support | Kamprad Family Foundation for Entrepreneurship, Research and Charity, Research and Development Primary Healthcare Regional Funds in Västra Götalandsregionen, Karolinska Institutet Funds, Capio Närsjukvård, Swedish Research Council, the regional agreement on medical training and clinical research (ALF), and Linnaeus University. |
| Primary sponsor | Linnaeus University |
| Secondary sponsor(s) | Karolinska Institutet |
| Contact for public queries | *AFVC* anneli.farnsworthvoncederwald@lnu.se |
| Contact for scientific queries | *AFVC* Department of Psychology, Faculty of Health and Life Sciences, Linnaeus University, Växjö, Sweden. |
| Public title | The KAIROS Project |
| Scientific title | Primary Care Behavioral Health in Sweden – A Cluster Randomized Trial Evaluating Outcomes Related to Implementation, Organization, and Patients (KAIROS) – Study Protocol |
| Countries of recruitment | Sweden |
| Health condition(s) or problem(s) studied | All mental and behavioral health issues commonly presenting in primary care |
| Intervention(s) | Experimental Arm: Direct implementation of Primary Care Behavioral Health (PCBH) Active Comparator Arm: Delayed implementation of PCBH, remaining in Care As Usual (CAU) during first study period |
| Key inclusion and exclusion criteria | Inclusion criteria: adult patient (≥ 18 years) who seeks primary care at participating unit, deemed to be suitable for behavioral health interventions Exclusion criteria: does not speak Swedish well enough to fill out questionnaires, is deemed to need emergency type care (such as immediate risk of suicide) |
| Study type | Interventional Allocation: cluster randomized Intervention model: parallel assignment Masking: double-blinded Primary purpose: treatment |
| Date of first enrolment | January 2022 |
| Target sample size | 595 |
| Recruitment status | Recruiting |
| Primary outcome(s) | Implementation level: Time until PCC has reached adequate PCBH model fidelity (fidelity level FID3) Organizational level: Waiting times until treatment Patient level: Daily functioning |
| Key secondary outcomes | Implementation level: Acceptability, feasibility and appropriateness of PCBH, obstacles to and facilitators of implementation Organizational level: Patient reach, treatment lengths, costs, interprofessional collaboration, staff work environment Patient level: Symptoms, satisfaction, subjective change, adverse events, treatment content, medication prescription, sick leave, care consumption |
