# Supplementary material for: Primary Care Behavioral Health in Sweden – a protocol of a cluster randomized trial evaluating outcomes related to implementation, organization, and patients (KAIROS)
Source: BMC Health Serv Res. 2023 Oct 31;23:1188. doi: 10.1186/s12913-023-10180-9 (PMC10619326; doi:10.1186/s12913-023-10180-9)
Supplement: Supplementary file 3 — Supplementary Material 3 [file 12913_2023_10180_MOESM3_ESM.docx]

**APPENDIX 2: MODEL CONSENT FORM**

The model consent form has been translated from Swedish.

**Information for Research Participants**

We would like to ask you if you would like to participate in the research project KAIROS. In this document, you will receive information about the KAIROS project and what it means to participate.

**What kind of project is this, and why do you want me to participate?**

Linnaeus University and Karolinska Institutet, in collaboration with [name of the PCC where the participant seeks care], are conducting the KAIROS project, which aims to investigate how primary care currently manages patients with mental and behavioral health problems and evaluate the effectiveness of treatments. The goal of the project is to improve the quality of psychosocial support and treatment in primary care.

We are asking you to participate in the study because you have sought care for your mental health, social situation, or a somatic condition that may be influenced by or affect your mental well-being. Please read this entire information. If you wish to participate in the study, you will provide your consent below.

**How does the study work?**

If you participate in the study, you will answer questions focused on your mental well-being, how you manage your daily life, and what areas you feel you need help with. You can answer these questions online or on paper, and it will take approximately 30 minutes. These questions can help you reflect on your difficulties. Your answers may also be viewed by the healthcare professionals you are in contact with, and they may choose to use them as an aid in selecting the appropriate treatment for you. Your answers will also help your healthcare center evaluate the effectiveness of the care provided.

After four, eight, and twelve weeks, as well as after one year, you will be asked to answer additional questions online or on paper regarding your well-being, any changes you have made, and your perception of the care received. Each time, it will take between 15 and 30 minutes to complete the questionnaire. We may also call you to gather additional information and ask about your experience with the care you have received. Long-term follow-ups of up to three years may be conducted, and if applicable, we will contact you. Your participation in the research study will not negatively impact your ability to access other healthcare services.

We will also collect data on sick leave, medications prescribed and bought, and healthcare utilization from the following public registers: Läkemedelsregistret (Swedish Prescribed Drug Register), Svenska Patientregistret (Swedish National Patient Register), MIDAS (Social Insurance Agency), PrimärvårdsKvalitet (Primary Care Quality) operated by Sveriges Kommuner och Landsting (Swedish Association of Local Authorities and Regions), and one of the regional registers VAL (Region Stockholm), SHR (Region Skåne), or VEGA (Region Västra Götaland) if you reside in any of these regions.

**What are the risks?**

The aim of the KAIROS project is to evaluate the care provided at the healthcare center, and therefore, you will receive the same care whether you participate in the project or not. No treatment will be withheld from you. However, answering questions in surveys and interviews may be perceived as burdensome as it takes around 30 minutes per occasion.

**Are there any benefits?**

The purpose of examining the treatments provided at the healthcare center is to improve the quality of psychosocial support and treatment in primary care. Ultimately, we hope that these benefits will benefit all patients in primary care. Currently, the main advantage of participating in the study is that the initial questions you answer can provide both you and your healthcare provider, if they choose to review your answers, with a better understanding of your situation and difficulties to make better decisions about your care.

**What happens to my data?**

The KAIROS project will collect and register information about you obtained from the questions you answer during the study, assessments made by healthcare professionals, medical records, and public registers. In order to conduct a scientific evaluation of each individual treatment, we will link this data to your personal identification number. Data will be stored digitally in a secure database controlled by the Primary Investigator during the study. Paper-based data will be stored in accordance with the healthcare center's procedures for patient information and then entered into the database. Once all the data in the study has been collected, it will be extracted from the secure database in pseudonymized form, where each participant is identified only by a code, and stored on a secure server at Linnaeus University. The key to the codes will be kept in the secure database to ensure that individuals cannot be identified during analysis. The results will be compiled statistically and presented in scientific journals and conferences without traceability to individual responses.

Healthcare professionals at your healthcare center involved in your treatment will have access to the answers you provide in the questionnaires, but they will not have access to data from public registers. If they choose to, healthcare professionals can use this information as a support in determining the most appropriate care for you. However, healthcare professionals decide for themselves whether they want to review your answers. Therefore, you should communicate directly with healthcare professionals about anything you want them to know about you and not use your responses in the KAIROS project as a means of communication with healthcare professionals. All information is processed in a way that unauthorized individuals cannot access it, and standard confidentiality rules apply. Data will be analyzed by participating researchers at Linnaeus University and Karolinska Institutet. Data will be stored throughout your lifetime to answer further research questions.

Linnaeus University is responsible for your personal data, which will be processed in accordance with the European Union's General Data Protection Regulation (GDPR) for research purposes, based on the legal basis of public interest. According to the GDPR, you have the right to access the information about you that is handled in the study, free of charge, and if necessary, have any errors corrected. You can also request the deletion of your data and restriction of the processing of your personal data. If you have any questions or want to access your data, please contact the Data Protection Officer at Linnaeus University at +46 470-76 75 78 or dataskyddsombud@lnu.se. If you are dissatisfied with how your personal data is handled, you have the right to lodge a complaint with the Swedish Data Protection Authority, which is the supervisory authority (datainspektionen@datainspektionen.se, +46 8-657 61 00).

**How will I receive information about the study's results?**

The results of the KAIROS project at the group level will be presented in scientific journals through Open Access, which means that the articles will be available for you to read when they are published.

**Insurance and compensation**

The study is conducted within regular healthcare, so no compensation or exemption from costs is provided, and regular patient insurance applies.

**Participation is voluntary**

Your participation is voluntary, and you can choose to withdraw at any time. If you choose not to participate or wish to withdraw, you do not have to state a reason, and it will not affect your future care. If you want to withdraw your participation, please contact the contact person for the KAIROS project. You can also communicate it directly to the members of the research team with whom you have direct contact, for example, during interviews.

**Contact person for questions about the KAIROS project and your participation:**

Anneli Farnsworth von Cederwald

Department of Psychology, Linnaeus University, 351 95 Växjö

Phone: +46 72 237 23 00, Email: anneli.farnsworthvoncederwald@lnu.se

**Research sponsor:**

The research sponsor refers to the organization responsible for the study. The research sponsor is Linnaeus University.

**Primary Investigator:**

Viktor Kaldo, Professor and Licensed Psychologist

Linnaeus University and Karolinska Institutet

Email: viktor.kaldo@lnu.se

**Consent to participate in the study:**

I have received oral and written information about the study and have had the opportunity to ask questions. I may keep/print/copy the written information.

☐ I consent to participate in the KAIROS project.

☐ I consent to the processing of my data as described in the research participant information.

Mobile number where we can reach you: _______________________________________________

Location and date: _________________________ Signature: _______________________________
